# A better start for health equity? Qualitative content analysis of implementation of extended postnatal home visiting in a disadvantaged area in Sweden

**DOI:** 10.1186/s12939-018-0756-6

**Published:** 2018-04-10

**Authors:** Madelene Barboza, Asli Kulane, Bo Burström, Anneli Marttila

**Affiliations:** 1 Equity and Health Policy Research Group, Department of Public Health Sciences, Karolinska Institutet, SE 171 77 Stockholm, Sweden; 2 Stockholm County Council, Centre for Epidemiology and Community Medicine, Box 45436, 104 31 Stockholm, Sweden

**Keywords:** Health inequity, Proportionate universalism, Nurturing care, Early childhood development, Extended home visiting, Qualitative study

## Abstract

**Background:**

Health inequities among children in Sweden persist despite the country’s well-developed welfare system and near universal access to the national child health care programme. A multisectoral extended home visiting intervention, based on the principles of proportionate universalism, has been carried out in a disadvantaged area since 2013. The present study investigates the content of the meetings between families and professionals during the home visits to gain a deeper understanding of how it relates to a health equity perspective on early childhood development.

**Methods:**

Three child health care nurses documented 501 visits to the families of 98 children between 2013 and 2016. A qualitative data-driven conventional content analysis was performed on all data from the cycle of six visits per child, and a general content model was developed. Additional content analysis was carried out on the data from visits to families who experienced adverse situations or greater needs.

**Results:**

The analysis revealed that the home visits covered three main categories of content related to the health, care and development of the child; the strengthening of roles and relations within the new family unit; and the influence and support located in the broader external context around the family. The model of categories and sub-categories proved stable over all six visits. Families with extra needs received continuous attention to their additional issues during the visits, as well as the standard content described in the content model.

**Conclusions:**

This study on home visiting implementation indicates that the participating families received programme content which covered all the domains of nurturing care as recommended by the WHO Commission on Social Determinants of Health and recent research. The content of the home visits can be understood to create enabling conditions for health equity effects. The intervention can be seen to represent a practical example of proportionate universalism.

## Background

Health inequities are systematically distributed in a social gradient within and between all societies [1]. The root causes can be summarized as “the unequal distribution of power, income, goods and services” [1]. To combat health inequities, the World Health Organization’s Commission on Social Determinants of Health (WHO CSDH) recommended policies of universal interventions which should be applied in higher dose and intensity to groups with higher levels of disadvantage and needs [1]. Marmot, the leader of the commission, in a subsequent English country report, named the theory proportionate universalism [2].

The same commission, in a specific report on early childhood development, pronounced that promoting a good start in life is priority for health equity [3]. Interventions focusing on the child and strengthening the caretaking capacities of the family are central, and evidence indicates that the most effective strategies are multisectoral and working through already existing service platforms in the community [3]. One such strategy is postnatal home visiting. Consistent evidence has been collected on the effectiveness of home visits in promoting early childhood development, improved parenting skills as well as positive effects later in childhood and adolescence [4–9].

### Health inequities in Rinkeby, Sweden

Despite having a well-developed welfare system, Stockholm, Sweden, is no exception to the workings of the social determinants of health. The County of Stockholm presents clear and increasing inequalities between geographical areas regarding socioeconomic vulnerability and health status [10–12]. Rinkeby, the location of the present study, is part of Rinkeby-Kista, one of Stockholm’s 14 districts. It is home to a diversity of immigrant groups, and where 91% of the population has foreign background (born outside Sweden or in Sweden with two foreign-born parents) [13]. Between 1991 and 2012, while the increase in average income for the population in the city centre was 64%, in Rinkeby-Kista it was 5%. Employment levels of 47% in Rinkeby can be compared to the highest district levels of over 85% in 2012 [12]. Many of the families with children in Rinkeby are exposed to social disadvantages, with 42% living in relative poverty (household income below 60% of the median income for families with children in the city of Stockholm) as compared to the city average of 12% in 2013 [12].

The children of Rinkeby present unfavourable health indicators from an early age, with 20% of new-borns exposed to tobacco smoke as compared to 10% in the county average in 2013, 15% of 3-year old’s having dental caries (4% county average), and 4% being obese at 4 years (2% county average). The coverage of measles, mumps and rubella vaccination at 18 months lay just below 82% while the county average reached 97% [11].

### The Rinkeby extended home visiting programme

The Swedish overall national goal for public health is to achieve the conditions for good health on equal conditions for the whole population [10], and although the child health care (CHC) program reaches almost all children in Sweden [11], these goals have not been achieved in areas such as Rinkeby. As a response, in 2013, Rinkeby CHC centre in collaboration with the social services developed an equity-based postnatal extended home visiting programme guided by the theory of proportionate universalism. All first-time parents were offered six home visits over 15 months, as compared to the one home visit recommended in the national CHC program. The visits were integrated in the universal CHC centre-based services. The intervention was carried out by teams comprised of a CHC nurse and parental advisor from the local social services.

The programme aimed at decreasing risk factors and increasing protective factors for the children’s health and wellbeing through the strengthening of the parents’ self-efficacy and health. Families’ increased integration into Swedish society through language learning and the child’s enrolment in public day care services were additional intended outcomes. The intervention was designed to involve existing local community services [14].

The programme content was based on the national CHC programme, structured around a health promoting theme for each visit [15]. The priority however, was to create an open meeting between families and professionals during which the parents’ needs and concerns would lead the intervention. The expectation was to build a trusting relationship where parents felt comfortable to engage in dialogue and ask questions. The importance of participation of fathers was also emphasized in the programme design [15].

A mixed-methods programme evaluation of the intervention, led by a team from Karolinska Institutet, has been ongoing since 2013. It includes questionnaires and interviews with parents and professionals, participant observation, analysis of child health records and records of health care utilization and document analysis [14]. Two reports have been published so far in Swedish [15, 16]. The present study was embedded in the programme evaluation and the content model presented in this article has previously been introduced in a master thesis at Karolinska Institutet by the first author.

### Relevance of the study

Despite the broad agreement on the importance of equitable early childhood strategies, the knowledge base is still weak on what interventions are actually effective [1, 17, 18]. Analysing how an intervention is implemented and the mechanisms that connect the inputs to the expected outcomes in a programme’s logical model is essential in the assessment of whether and why the goals are achieved [19]. An additional important aspect when studying health inequities is to investigate the theories of proposed interventions to understand why they might work [20]. Still, the implementation process and programme content have been rare features in evaluations of home visiting. Previous research emphasize the need for documenting and understanding home visiting intervention mechanisms [21–23]. To our knowledge, no scientific study has yet been conducted of the implementation of an equity-based extended postnatal home visiting program for first-time parents, guided by proportionate universalism and carried out in collaboration between CHC services and social services.

The aim of this study was to investigate the content of the meetings between families and professionals during the home visits and gain a deeper understanding of how it relates to the concepts of proportionate universalism and equitable early childhood development.

## Methods

The present study investigated the implementation of the Rinkeby extended home visiting programme through a qualitative content analysis of the CHC nurses’ documentation of visits. A qualitative research approach was chosen to enable the description of human experience, interaction and principles, and to develop new concepts and theoretical models [24].

### Material

A total of 119 children were registered at Rinkeby CHC centre during the inclusion period (01.09.2013–31.08.2014). Of those, 11 moved away from the area soon after birth. From the remaining group, the families of 101 children (94%) gave consent to and participated in the intervention and evaluation study [15].

For the analysis of home visiting documentation, all families who had participated in 2 or more of the planned 6 visits were considered, which led to the final inclusion of 98 children. A total of 501 visits took place to these families and were documented between September 2013 and March 2016. This represents 84% of the programme’s planned visits. The distribution of visits received by the children and parents is depicted in Table 1. An additional seventh visit was received by 4 children.

**Table 1:** Family participation in the Rinkeby extended home visiting programme

Number of visits received for each child	Number of children (*N* = 98)
2	6
3	7
4	8
5	30
6 or 7	47

Many families experienced housing instability and parents were not able or were reluctant to receive the professionals in their temporary accomodations. The option was therefore given to carry out the visit at the CHC centre and this happened in 22% of the visits.

### Participants

The participants consisted of a diverse group where 8 mothers were born in Sweden while the rest came from 30 different countries. The largest national group consisted of 39 mothers from Somalia. Most had migrated for reasons of work or family reunification. A small group were asylum seekers and a few were undocumented and living in hiding. Nearly half (46%) had lived in Sweden for 3 years or less and 27% lived in temporary accommodations. Schooling levels were 8 years or less for 39% of the mothers, 9–12 years for 32%, and 13 years or more for 29%. One third of them were living without a partner [15]. Still, the families were also represented by the fathers, 79% of whom participated in at least one visit. A total of 40% of the visits counted on fathers’ presence.

### Data collection

The data of home visiting implementation was registered by the three CHC nurses who participated in the home visiting teams. Specific templates were used for the documentation of each visit. The templates had assigned spaces for notes on topics that were expected to be discussed, for example “feeding” or “child safety”, as well as spaces for parents’ questions and concerns, and open spaces for notes on any other issues that were discussed. The nurses filled out the templates immediately after each visit. The templates were developed by the programme evaluation coordinator from Karolinska Institutet in collaboration with the CHC nurses. The documentation was monitored regularly through sessions of supervision given to the nurses by the evaluation coordinator. Issues and doubts regarding the process of registering the visits were then discussed and handled.

### Analysis

A data-driven conventional content analysis [25] was selected as the analytical approach, using the terms: *code*, *sub-category* and *category*. The analysis focused on the main part of the text, which represented the communication and interaction between professionals and parents (and in a few cases, other caretakers such as grandparents). The documentation also contained a few additional notes, for example concerning the presence and actions of interpreters, or the nurses’ own thoughts regarding the actions of the parental advisors. These parts of data were excluded from the analysis.

#### Home visits 1–6

The documentation was sorted into 6 groups, so that visits 1 to all families were analysed together, followed by visits 2 and so on. The documentation of the first visit to all families was analysed by assigning codes to the content, followed by the creation of sub-categories. Codes and sub-categories were discussed and reviewed with the research team and organized into tentative categories. The same procedure of coding was repeated with home visit 2, applying new codes to the content where necessary. The organizing of codes proved very similar to that of visit 1, and a content model with more robust sub-categories and categories started to take form. From visit 3 onwards, the starting point for coding continued to be the data, while the framework of sub-categories and categories was kept, although reviewed along the process of analysis. Having completed analyses of the 6 groups, descriptions of the content of each home visit was produced and the final model of sub-categories and categories was defined. A joint analysis of the 6 visits as a process was then carried out in order to identify patterns and development of the content over time.

#### Frequency count

Frequency counts that summarize themes and categories have been recognized for their usefulness during parts of the qualitative analysis [26], and also as a strategy to ensure the reliability of the analysis [27]. Therefore, a frequency count of sub-categories was carried out for each home visit and contributed to the analysis of visits 1–6 as a process.

#### Families with extra needs

At this point in the study it was considered useful to include an additional step of analysis to investigate whether there were any specific characteristics of the content of visits to families where additional needs, adversities or problematic issues were present. During a new reading of all original data, purposeful sampling was carried out and the documentation of visits to 18 representative families were identified. The cycle of 6 visits to each of the selected families was analysed and comparative analysis was carried out within the group of families to identify general patterns and characteristics. These, in turn, were compared to the standard content model and patterns for the families of all 98 children.

All steps of analysis and products were reviewed by the research team.

## Results

### Content model

**Fig. 1:**
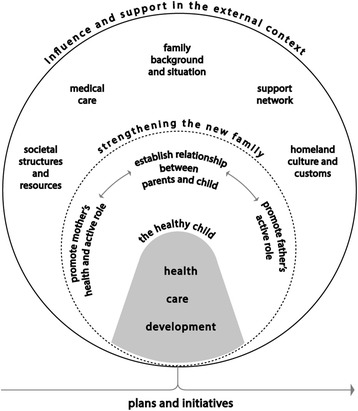
Model of implemented content of home visits in the Rinkeby extended home visiting programme

The content analysis of the CHC nurses’ documentation of six home visits produced a model of three main categories encompassing eleven sub-categories, presented in Fig. 1. A twelfth sub-category emerged during the home visiting process, assuming a prominent role only in the last visit.

*The healthy child* is the central category in the content of communication during all six home visits. The child focus is introduced at the start of each visit by the parents’ describing their own observations of the child’s progress since the last meeting, followed by child-centred information by the professionals. The sub-categories of *health*, *care* and *development* represent the three aspects directly concerning the promotion of the healthy child. *Health* deals with the child’s physical growth and well-being, prevention and treatment of illness. *Care* regards the routines of feeding and nutrition, sleeping patterns, hygiene and safety. *Development* includes issues of love, interaction, stimulation, play and language. *Health, care* and *development* are interrelated and complementary to one another in the communication between parents and professionals. Often all three sub-categories are present together in the same conversation.

*“Works better with sleep, has stopped breastfeeding. Mother has questions about diarrhoea and vomiting. Questions about food and how to do – he doesn’t want to eat normal food. Needs to put music on. Talk about that he is active. Advice about open preschool in the new neighbourhood.” (Home visit 5)*

*Strengthening the new family* is the category that surrounds the child in the model and it encompasses the sub-categories of *promote mother’s health and active role*, *promote father’s active role* and *establish relationship between parents and child*. The latter sub-category, encompasses the three-way relationship between mother-father, mother-child and father-child. To form a protective and supportive environment for the child’s development through the strengthening of the family is given continuous attention during the course of visits.

*“Development, mother and father have learnt the child’s signals, know immediately what he wants.” (Home visit 2)*

*“Mother and father – good relation, help each other.” “Relation to the child – play a lot with him, eat together, have fun.” (Home visit 3)*

Often the relationship between parents and child is brought into the conversation together with content concerning the child’s development, where the importance of the emotional bonding, interplay and interaction are recurrently stimulated and valued by the professionals.

*“The brain develops when mother communicates with the girl. We talked about what the child understands, signals. Development, what will happen from now on.” (Home visit 3)*

*“Looks at father when she says father, looks at mother when she says mother. We talk about reading books, hand out the picture book, father shows the girl and tells the story in the book.” (Home visit 4)*

The sub-category of *promote mother’s health and active role* is most prominently present during the first visit and regards the mothers’ health after giving birth, when special attention is given to detecting post-partum depression.

*“Talk about mother’s and father’s feelings. Delivery – still feels pain. Advice on how to make breastfeeding work – mother wonders how to do. I am often sad – cry, tells about stress and how she handles it.” (Home visit 1)*

*Promote father’s active role* is a sub-category especially acknowledged in the content and it can be observed how the fathers are intentionally mentioned by the professionals during the visits, even when absent. There is repeated motivation to make the fathers participate in the visits when possible.

*“Child cries when we arrive. Is tired. Father makes her go to sleep after a while. Acknowledgement and encouragement around this.” (Home visit 2)*

The outer layer of the content model represents the category of *influence and support in the external context*, and is made up of diverse elements in the surroundings that exercise influence over the families or provide support to them. The sub-category of *family background and situation* mainly contains content related to circumstances that exert influence over the family at present, such as housing situation, migrant status or the family structure around the child. It also includes content on parents’ backgrounds and reflections on their own upbringings. The content in this sub-category is sometimes of a sensitive nature, revealing complex and difficult family issues. On some occasions, this category contains direct requests for help.

*“Can you help us find accommodation? Can only stay another week in the apartment.” (Home visit 2)*

The sub-category of *support network* contains conversation around existing networks of family and friends that relieve the parents from the daily load of child care. Some content of mothers’ feelings of loneliness and isolation is also registered. This sub-category is promoted by the professionals through their continuous encouragement to the mothers to get out of the house and participate in activities where one can meet other mothers.

*“Conversation around that mother seems downhearted – mother feels lonely and “bored”, talk about this. Recommend going to open preschool or parent and baby Swedish classes. Important to go out.” (Home visit 4)*

The *homeland culture and customs* sub-category introduces content of cultural diversity and sometimes of tension, when parents find themselves torn between child care guidance given by the professionals and conflicting advice from relatives. These situations leave the parents pressured to decide whether to adopt the Swedish way or follow cultural traditions from their home country.

*“Is it ok to give a little olive oil in the formula? It helps the stomach. Someone has said it is dangerous. Grandmother gives tap water and also sugar on the pacifier, is it ok?” (Home visit 2)*

The sub-category of *societal structures and resources* is often actively introduced into the visits by the professionals, and concerns resources such as the open preschool, parenting groups or parent and baby Swedish classes. These resources serve multiple purposes of offering activities for the parents and children, creating support networks, as well as opening doors for integration into Swedish society.

*Medical care* contains the professionals’ general information to parents on how, where and when to seek health care for the baby. It is recurrent in cases of a child’s medical needs, when the professionals refer parents to the correct service provider in the health care system.

The twelfth sub-category of *plans and initiatives*, however, emerged in the documentation of content only towards the last visits. Mostly related to the parents’ wishes and actions to start studying, working or find better housing, it had a more prominent presence at the last encounter when the parents were asked to picture their life in 1 year’s time.

### The process of 6 home visits

The analysis of the 6 visits as a process revealed similar patterns of content throughout the whole cycle. The codes varied somewhat from one visit to another, following the process of development of the child, but the 11 main sub-categories and 3 categories in the content model remained stable. While primary focus regarded the child and the relationship between parents and child, content from all three categories was continuously present in the texts from each visit. The frequency count of sub-categories carried out on all data supported the observation of stability of the content model. All 11 main sub-categories were observed at relatively constant levels over the course of six visits. The highest overall frequencies were observed in the sub-categories of *health, care, development* and *establish relationship between parents and child*, reinforcing the centrality of the categories of *the healthy child* and s*trengthening the new family.*

### Families with extra needs

Following the main analysis, the supplementary analysis of a sub-sample of 18 families experiencing specific needs or adverse situations, revealed some specific aspects in terms of content. The special issue or need experienced by the family was discussed and dealt with during the visit, while the professionals also covered all content in the general content model. A degree of flexibility was applied in some cases, giving priority during the visit to the specific issue that affected the family, or booking extra meetings with the family to deal with that particular situation. The issue or situation was then continuously followed up during all subsequent visits and the parents were given space to talk about their concerns. The professionals appear to balance the difficult and negative content with positive issues during the visits to these families, often by introducing contents related to the child that were generally concrete and joyful.

*“Show love: Conversation about nice moments and encouragement around this. Talk about the relationship and how they handle it. Conversation around food and breast feeding. Routines. Information: Book and the importance of reading. Toothbrush and toothpaste. Information/talk around handling anger and how raised voices may affect the child. [...] Own questions: conversation around father’s anger. Around how they still experience difficulties with accommodation and the social services.” (Home visit 4)*

A few of the families encountered very serious adversities, such as decisions regarding deportation or eviction notices, and the home visits would then predominantly deal with the crisis, leaving little time for the standard content of the model.

## Discussion

The present study investigated the content of implementation of the Rinkeby extended postnatal home visiting programme from a health equity perspective.

### Nurturing care for equitable early childhood development

The content analysis produced a model which was centred around the child with three levels of interrelated content that focused on the direct care of the child, the strengthening of the family unit and the external factors that influenced and supported the families. This content model appears to correspond well to the recommendations made by the WHO CSDH [3] and the third Lancet series on child development [4, 28, 29], that interventions should consider individual and family, as well as community and society spheres. The inner levels of child and family environments in the content model contain the essential components of nurturing care defined by Britto et al. in the Lancet series as: “caregiving (e.g. health, hygiene care, and feeding care); stimulation (e.g. talking, singing, and playing); responsiveness (e.g. early bonding, secure attachment, trust, and sensitive communication); and safety (e.g. routines and protection from harm)” [29].

In the theory of nurturing care, a central role is given to the family unit as provider of care and a nurturing environment, rather than targeting only mother and child [3, 29]. The category of *strengthening of the new family* in the content model seems to correspond to similar assumptions. It contains content that promotes the three-way relationship between mother-child, father-child and mother-father, as well as specific focus on strengthening the role of each parent. Irwin et al. consider that the role of fathers in providing nurturing care is often overlooked [3], but this proved to be an important component in the Rinkeby home visiting programme’s implementation. The presence of fathers was specifically noted in the content and recurrent motivation for their participation was given.

Outside of the family unit, the surrounding community and network are viewed as important in providing resources and support to parents’ nurturing practices [3, 29]. The content model’s outer layer indicates the presence of these aspects also in the Rinkeby home visiting programme. The professionals’ referrals to specialized services when needed and their continuous recommendation of open preschool and other local services indicate that the programme has acted as a channel of access for the families to the wider early childhood service network.

### Proportionate universalism in practice

The extended home visiting programme was planned as an intervention of proportionate universalism and although the principles of proportionate universalism have been widely recognized, there is still little clarity on the mechanisms by which this approach could be effectively implemented within universal services [30, 31]. Cowley et al. suggest that postnatal home visiting can serve well as such a strategy [31]. It constitutes a platform for delivering universal services while also providing support for those in higher need through mechanisms such as needs assessment of the family, flexible visiting schemes, intensive support, indications, referrals, motivation and support in accessing additional services and resources [31]. These mechanisms could also be observed in the content analysis of the Rinkeby extended home visiting programme. The 6 home visits offered repeated exposure to key contents of the national programme, which gave the possibility of returning to specific contents to provide extra support or to motivate parents to access other services. Flexibility was also observed in the programme implementation, with time for parents’ questions and own contributions, as well as the scheduling of extra visits where necessary. Those families who experienced adversities, received specific attention to their need while still having access to the same general home visiting content as the rest of the group.

### Barriers to programme uptake

The analysis of the implementation of the Rinkeby home visiting programme showed that it contained content which corresponds to the principles of proportionate universalism and equitable early childhood development. However, other factors of family participation and uptake of content often represent barriers in home visiting [21, 23] which may negatively affect conditions for health equity outcomes. Low enrolment and high drop-out rates are commonly reported in home visiting interventions, especially among high-risk groups [21, 32]. However, he comparatively high levels of family participation in the Rinkeby programme, both in terms of enrolment and number of visits received by each family (Table 1), suggests that this has not been the case. The high levels of participation of fathers as well, further corroborate this observation.

Housing insecurity has also been observed as a barrier to uptake of programme content [23, 33] as well as situations where families’ urgent needs or crises have hindered implementation of the foreseen content of interventions [23, 34]. These barriers were also observed in the Rinkeby programme, in relation to families who experienced adversities. Many families had difficulties in receiving visits due to housing instability. For this reason, the programme design allowed for the possibility of carrying out the visits at the CHC centre. This happened in 22% of all visits and may have contributed to securing the participation of some families with greater needs. Still, in a few families, severe crisis situations, negatively affected the delivery of content in comparison to the standard content model.

### Implications for practice and further research

The third Lancet series on child development calls for the scaling up of evidence-based interventions to combat global health inequity [28]. Multisectoral intervention packages based on nurturing care are recommended, and the Rinkeby extended home visiting programme would represent an example of a “family support and strengthening package” [29].

However, the implementation of intervention packages of nurturing care depends on supportive socioeconomic and political contexts which include policies and legal and organizational structures and systems [28]. The programme in Rinkeby was set within the Swedish network of welfare policies and early childhood health, education and social protection services on municipal, regional and national levels, and the presence of this network was frequently visible in the home visiting content. Maggi et al. discuss the need for understanding of the causal relations between macro social policy, other intermediate determinants and early childhood development [35]. The present study provides speculation rather than robust evidence on this aspect. However, it does seem plausible to suggest that without the wider level policies and resources in place, the complexity of the home visiting content model would be reduced and probably also limit the potential for positive effects. This could thus have implications for scaling up of the Rinkeby model to settings with fewer resources on the macro level. Therefore, further research needs to be carried out on equity-based home visiting models in contexts where structures and services are reduced or absent.

### Limitations

The main limitation of this study relates to the documentation being produced by the CHC nurses themselves rather than through on-site observation by a professional researcher. However, measures were taken to ensure that the data was suitable for qualitative analysis. The CHC nurses participated in the development of templates for documentation of visits and they also received continuous support and supervision by the evaluation coordinator. The findings of the analysis of the CHC nurses’ documentation were compared to findings from questionnaires and in-depth interviews with parents that were part of the overall programme evaluation. The parents’ descriptions of the home visiting content correspond well to the CHC nurses’ documentation and no element was mentioned by the parents that was not present in the documentation. This strengthens our belief that the CHC nurses’ documentation was a credible data source for qualitatively analysing the content of the home visits.

## Conclusions

This study on home visiting implementation indicates that the participating families received programme content which covered all the domains of nurturing care as recommended by the WHO CSDH and recent research. The content of the home visits can be understood to create enabling conditions for health equity effects. The intervention can be seen to represent a practical example of proportionate universalism.

## Abbreviations

CHC: Child health care
WHO CSDH: World Health Organization Commission on Social Determinants of Health
